# A Vicious Cycle: In Severe and Critically Ill COVID-19 Patients

**DOI:** 10.3389/fimmu.2022.930673

**Published:** 2022-06-15

**Authors:** Peifeng Huang, Qingwei Zuo, Yue Li, Patrick Kwabena Oduro, Fengxian Tan, Yuanyuan Wang, Xiaohui Liu, Jing Li, Qilong Wang, Fei Guo, Yue Li, Long Yang

**Affiliations:** ^1^ School of Integrative Medicine, Tianjin University of Traditional Chinese Medicine, Tianjin, China; ^2^ School of Department of Clinical Training and Teaching of Traditional Chinese Medicine, Tianjin University of Traditional Chinese Medicine, Tianjin, China; ^3^ State Key Laboratory of Component-Based Chinese Medicine, Tianjin University of Traditional Chinese Medicine, Tianjin, China; ^4^ National Health Commission of the People’s Republic of China Key Laboratory of Systems Biology of Pathogens, Institute of Pathogen Biology and Center for AIDS Research, Chinese Academy of Medical Sciences & Peking Union Medical College, Beijing, China; ^5^ Research Center for Infectious Diseases, Tianjin University of Traditional Chinese Medicine, Tianjin, China

**Keywords:** COVID-19, PAI-1, IL-6, inflammatory reaction, venous thrombosis, tocilizumab, endothelial cells

## Abstract

The coronavirus disease 2019 (COVID-19), caused by the severe acute respiratory syndrome coronavirus 2 (SARS-CoV-2) virus, is one of the fastest-evolving viral diseases that has instigated a worldwide pandemic. Severe inflammatory syndrome and venous thrombosis are commonly noted in COVID-19 patients with severe and critical illness, contributing to the poor prognosis. Interleukin (IL)-6, a major complex inflammatory cytokine, is an independent factor in predicting the severity of COVID-19 disease in patients. IL-6 and tumor necrosis factor (TNF)-α participate in COVID-19-induced cytokine storm, causing endothelial cell damage and upregulation of plasminogen activator inhibitor-1 (PAI-1) levels. In addition, IL-6 and PAI-1 form a vicious cycle of inflammation and thrombosis, which may contribute to the poor prognosis of patients with severe COVID-19. Targeted inhibition of IL-6 and PAI-1 signal transduction appears to improve treatment outcomes in severely and critically ill COVID-19 patients suffering from cytokine storms and venous thrombosis. Motivated by studies highlighting the relationship between inflammatory cytokines and thrombosis in viral immunology, we provide an overview of the immunothrombosis and immunoinflammation vicious loop between IL-6 and PAI-1. Our goal is that understanding this ferocious circle will benefit critically ill patients with COVID-19 worldwide.

## Introduction

Since late December, coronavirus disease 2019 (COVID-19) (1) has spread worldwide and instigated a pandemic. Globally, as of April 12, 2022, more than five hundred million people have been diagnosed with COVID-19 disease, including more than 6 million deaths from the disease (WHO, https://covid19.who.int/), posing a great challenge to the health system around the world. The causative agent of the disease is the SARS-CoV-2 virus. Based on the clinical presentation of the COVID-19 disease, the mild-to-moderate disease accounts for 81% of COVID-19 infections and is accompanied by symptoms such as cough, fever, fatigue, and others. Meanwhile, only about 14% of cases have severe symptoms such as dyspnea and hypoxemia, while 5% present with respiratory failure, shock failure, multiple organ failure, and other severe conditions that can result in death. In addition, 14.8% of patients are classified as severe or critically ill patients (**Table 1**) **(** 2). Emerging laboratory and pathological examination data indicate that cytokine storms and thrombosis were closely related to the disease progression, accounting for the poor prognosis in COVID-19 patients (3–8).

**Table 1:** The distribution of age, degree, and fatality rate of COVID-19 (2).

Categories	Subgroup	Cases	Distribution
Age	≥80 years	1,408	3%
	30–79 years	38,680	87%
	10–29 years	4,168	6%
	<10 years	416	1%
Degree	Mild	36,160	81%
	Severe	6,168	4%
	**Critically ill**	**2,087**	**5%**
Fatality rate	44,672 confirmed cases	1,023	2.3%
	Aged ≥80 years	208	14.8%
	70–79 years	312	8.0%
	**Critically cases**	**1,023**	**49.0%**

Bold values highlight the proportion and mortality of critically ill patients and emphasize the lethality of COVID-19.

**Figure 1:**
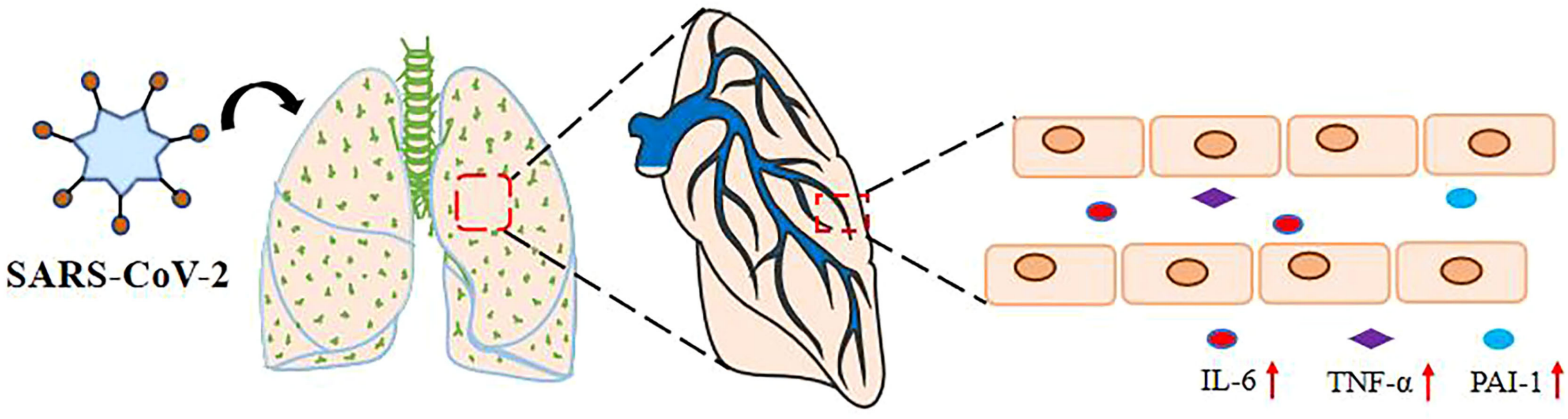
SARS-Co-2 upregulates plasma IL-6, TNF- α, and PAI-1 levels. The levels of IL-6, PAI-1, and TNF-α in the serum of severely and critically ill COVID-19 patients with SARS-CoV-2 pulmonary infection *via* the respiratory tract were significantly increased.

A significant reduction in spontaneous clot dissolution after activation of the external clotting pathway and increased resistance to tissue plasminogen activator (tPA) suggests a potential link between fibrinolytic disorder and thrombosis (9). Serum proteomics studies in patients with COVID-19 have found that abnormal increases in IL-6 correlate with increases in the coagulation and complement cascade components (10). PAI-1 is a serine protease inhibitor that acts as a principal inhibitor of tPA and urokinase-type plasminogen activator (uPA) to inhibit fibrinolysis. Based on PAI-1’s primary function, diseases, or disorders that increase PAI-1 levels appear to result in high coagulation states (11–13). Interestingly, in patients with mild-to-moderate disease, plasma levels of PAI-1 were normal compared to critically ill COVID-19 patients (14, 15). However, reports from studies suggested that PAI-1 levels significantly increase in critically ill (14) and hospitalized COVID-19 patients (**Figure 1**). In addition, previous analyses on the detection of inflammatory and prethrombotic biomarkers in the blood showed significant differences between IL-6 and PAI-1 levels. The mean concentration of IL-6 in the non-severe COVID-19 group was 430.3 pg/ml, whereas that of the control group was 419.5 pg/ml. Meanwhile, the concentration of IL-6 in severe COVID-19 and death group was 1,463 and 2,200 pg/ml, respectively (14). PAI-1 is a widely recognized biomarker of endothelial dysfunction and has been shown that increased concentration is associated with the severity of the disease (16, 17). The expression of PAI-1 may reflect the severity of SARS-CoV-2 infection to some extent (18). The plasma concentration of PAI-1 detected in patients with severe COVID-19 was 713.3 ng/ml, while in the COVID-19 death group, it was 1,223.5 ng/ml. Then again, in the non-severe COVID-19 group, the plasma concentration of PAI-1 was 465.2 ng/ml and that of healthy donors was 183.7 ng/ml (14). It is important to note that severe and critically ill patients with COVID-19 often suffer from underlying diseases (19, 20). Evidence has also suggested that most of the underlying diseases present with elevated levels of PAI-1 (21). For example, among diabetes and acute cerebral infarction patients without COVID-19, PAI-1 levels averaged 36.5 and 63.95 ng/ml (22, 23). Nonetheless, COVID-19-infected individuals have significantly higher levels of PAI-1 than those with diabetes or acute cerebral infarction, providing indirect evidence that COVID-19 could increase PAI-1 levels (**Table 2**).

**Table 2:** The expression of IL-6 and PAI-1 in COVID-19 and underlying diseases.

Disease		IL-6 (mean pg/ml)	PAI-1 (mean ng/ml)
COVID-19	Healthy donors	419.5	183.7
	Non-severe COVID-19 group	430.3	465.2
	Severe COVID-19 group	1463	713.3
	Death group	2200	1,223.5
Type 2 diabetes	–	<20 (24)	36.5
Acute cerebral infarction	–	<1,000 (25)	63.95

Studies on coexpression-induced IL-6 and PAI-1 through the nuclear factor-kappa B (NF-κB) pathway and ligand-dependent epidermal growth factor receptor (EGFR) activation confirmed a significant correlation between IL-6 and PAI-1 (26). The same phenomenon has revealed significant differences between IL-6 and PAI-1 levels in severe and mild-to-moderate COVID-19 patients (14). Treatment with anti-TNFs can reduce the death rate and poor outcomes of COVID-19 patients (27). Below, we review the possible relationship between inflammatory levels and thrombosis in severe and critically ill COVID-19 patients.

## SARS-CoV-2 Raises the Expression of PAI-1, IL-6, and TNF-α

The SARS-CoV-2 infection has a devastating effect on immune regulation, leading to a life-threatening systemic inflammatory syndrome called the cytokine storm. This systemic inflammatory syndrome involves abnormal immune-cell hyperactivation and uncontrolled release of circulatory cytokines. Elegant evidence from the COVID-19 pandemic shows that IL-6 and TNF-α are involved in the COVID-19-induced cytokine storm (28). In severe disease, IL-6 and TNF-α are major contributing factors that worsen the condition and cause poor clinical outcomes and even death (29–31). IL-6 is a multifunctional cytokine capable of transmitting cell signals. It is the main trigger of endothelial cytokine storm and an intervention target for clinical therapy (32, 33). Almost all stromal cells and immune system cells can produce IL-6, and the primary activator is IL-1β or TNF-α (34). Toll-like receptor (TLR)-stimulated monocytes and macrophages can also promote the expression of IL-6 (35).

During propagation of the SARS-CoV-2 virus, the envelope spike glycoprotein of the SARS-CoV-2 virus attaches to the angiotensin-converting enzyme (ACE)-2 on the target cell surface, resulting in ACE-2 loss (36). ACE-2 is a negative regulator that functions by activating tPA. ACE-2 deficiency disrupts the effective ACE-2/angiotensin (1–7)/Mas receptor axis, making Ang II more active and decreasing tPA activity, prompting endothelial and smooth muscle cells to synthesize and release PAI-1, leading to the balance of PAI-1/tPA to revert to its prethrombotic state (37, 38). Studies on intensive care unit (ICU) patients with critically ill COVID-19 found that low fibrinolysis was mainly associated with elevated PAI-1 levels (39). The action of recombinant SARS-CoV-2 on the ACE-2 receptor is comparable to that of live viruses, and its spiking glycoprotein induces the expression of PAI-1 in human pulmonary microvascular endothelial cells (HPMECs) (40). In individuals with severe COVID-19 illness, increased PAI-1 expression reduces tPA activity and increases thrombosis while perhaps worsening the inflammatory response (**Table 3**).

**Table 3:** The expressions of PAI-1 and IL-6 in severe COVID-19 patients.

Factors	Expressing and working	Reference
PAI-1	rSARS-CoV-2-S1 infect HPMECs exhibited robust induction of PAI-1	(40)
	Circulating levels of PAI-1 upregulate and function as an independent predictor of the severity of COVID-19 disease in patients	(41)
	Decreased the PAI-1 levels and alleviated critical illness in severe COVID-19 patients	(42)
	Significant expression of PAI-1 exists only in severe COVID-19 patients and promotes patient thrombosis	(14)
	Hypercoagulability and hypofibrinolysis are connected to the elevated level of PAI-1 in COVID-19	(39)
IL-6	IL-6 can serve as an independent factor predictor of the severity of COVID-19 disease in patients	(43–46)
	Seroproteomics studies found IL-6 significant upregulation, and IL-6 signal transduction is the most upstream upregulation pathway in severe patients with COVID-19 patients	(10)
	IL-6 is the main trigger of endothelial cytokine storms in COVID-19 patients	(32)

### PAI-1 Upregulates the Expressions of IL-6 and TNF-α

In several studies, PAI-1 has been found at the inflammatory site after tissue damage (47, 48). PAI-1 inhibitors reduce TNF-α expression and, at the same time, decrease PAI-1 expression in diabetic mice (49). PAI-1 upregulation may be related to its capacity to activate macrophages. PAI-1 helps to regulate the lipopolysaccharide (LPS)-induced inflammatory response in NR8383 cells, possibly by influencing the TLR4-myeloid differentiation protein 2 (MD-2)/NF-κB signaling transfer pathway (50). PAI-1-induced TLR4 activation causes monocyte macrophages to release significant quantities of IL-6 and TNF-α, exacerbating the inflammatory response (51, 52). This shows that TLR4 is an essential medium for PAI-1 to activate macrophages and promote TNF-α expression. The expression spectrum of macrophages stimulated by PAI-1 occurs 2 h after the peak transcription of PAI-1 (53). PAI-1 can promote macrophage activation and may also be an initial response gene for predicting inflammation. PAI-1 promotes the recruitment of monocytes/macrophages in tumor cells. Its lipoprotein-receptor-related protein 1 interaction domain regulates macrophage migration, whereas its C-terminal uPA interaction domain auto-secretes IL-6 by activating the p38MAPK and NF-κB pathway and inducing macrophage polarization (54). There was a considerable increase in the expression of M1 macrophages in obese mice caused by a high-fat diet (HFD), but PAI-1 deficiency and PAI-039 therapy prevented the development of these markers, demonstrating that PAI-1 is required for macrophage polarization. Meanwhile, PAI-1 activates TLR4, triggering a robust inflammatory response in endothelial cells (ECs), allowing ECs to continuously secrete IL-6 (55). PAI-1 may interact with TLR4 to activate NF-κB, leading ECs to generate cytokines such as IL-6 (56, 57). This shows that PAI-1 can stimulate macrophages and endothelial cells in various ways, promoting inflammatory responses (**Table 4**).

**Table 4:** PAI-1 upregulate the expressions of IL-6 and TNF-α.

Targets	Cell/host	Model	Mechanism	Reference
PAI-1 upregulates TNF-ɑ	NR8383 cells	Inflammatory model induced by LPS	TLR4-MD-2/NF-κB signaling transduction pathway	(50)
	Mouse	Type 2 diabetes mellitus	PAItrap3 decreases the levels of both PAI-1 and TNF-α	(49)
	Mouse	Systemic inflammation model	PAI-1 regulates inflammatory responses through TLR4 mediated macrophage activation	(53)
PAI-1 upregulates IL-6	C57 mouse/HT-1080 fibrosarcoma cancer cell line	Rag1^−/−^ PAI1^−/−^/Rag1^−/−^PAI-1 mice	PAI-1 promotes the recruitment and polarization of macrophages in cancer	(54)
	Microvascular (MIC) and macrovascular (MAC) endothelial cells (ECs)	Inflammatory model induced by LPS	PAI-1 was necessary for macrophage polarization	(55)
	Mice/human aortic endothelial cells (HAECs)	Endotoxemia of mouse/Inflammatory model induced by LPS	PAI-1 combines with TLR4 to promote NF-κB activation so that ECs produce chemokines, such as IL-6	(56, 57)

There is no clinical use of PAI-1 inhibitors in COVID-19 patients. However, it is worth noting that bortezomib upregulates KLF2 to suppress PAI-1 expression and reduce EC damage in HPME cells stimulated with rSARS-CoV-2-S1 glycoprotein (58).

### The IL-6 Increases the Expression of the PAI-1

Severe clotting disorder in patients with COVID-19 is closely related to the increased risk of death (59–62). Venous thromboembolism was prevalent in COVID-19 patients, with a total incidence of 31% in 184 patients with severe COVID-19 (63), and a preliminary autopsy on 11 of the COVID-19 patients revealed thrombus in the pulmonary arterioles (64). The D-dimer is a fibrin degradation product used as an alternative marker of fibrinolysis and is often elevated in thrombotic events (65). Relevant studies on COVID-19 report that D-dimer elevation is a prevalent feature (66). Low fibrinolysis is the primary cause of increased blood viscosity and is associated with elevated PAI-1 levels (39). PAI-1 circulating levels may be used as an independent predictor of severity in COVID-19 patients (41), and regulating PAI-1 expression can benefit patients with COVID-19 (42).

Alongside PAI-1, IL-6 is an independent predictor of COVID-19 severity (43–46). IL-6 levels have a substantial predictive value for mortality in COVID-19 ICUs (67). Patients with severe COVID-19 have considerable IL-6 overexpression, and IL-6 signal transduction is the most upregulated pathway in COVID-19 patients (10). IL-6 may have a significant role in the progression of severe COVID-19 disease in patients. PAI-1 expression is only found in severe COVID-19 patients and increases thrombosis (14). PAI-1 is linked to elevated levels of IL-6 in critically ill COVID-19 patients. IL-6 signals through two central pathways. The first is the classic cis signaling, and the second is the trans-signaling. In the classic cis pathway, IL-6 attaches to cells, mainly immune cells, expressing the membrane-bound interleukin-6 receptor (IL-6R) to initiate a downstream signaling response (68, 69). On the other hand, in trans-signaling, IL-6 binds to the soluble form of IL-6R, which is released from IL-6R expressing cell surfaces by proteolysis and IL-6R mRNA to form an exciting complex that associates with membrane-bound gp130 (70–72). In the presence of high circulating levels of IL-6, trans-signaling typically occurs. For instance, ECs express the membrane-bound gp130 but not the membrane-bound IL-6R (73–76), allowing for IL-6/soluble-IL-6R/gp130 downstream signaling activation.

The detection of PAI-1 expression before and after tocilizumab (TCZ) treatment demonstrates that IL-6 signaling transduction can promote PAI-1 expression in ECs (18, 42). LPS stimulates the NF-κB classical pathway to increase the PAI-1 expression and promote alveolar hypercoagulation and fibrinolysis inhibitory states. PAI-1 expression is dramatically reduced following NF-κB knockout (77, 78), indicating that the NF-κB pathway can control PAI-1 expression to some extent. At the same time, elevated plasma IL-6 levels promote NF-κB activation (79), resulting in EC-induced PAI-1 overexpression. In hepatocytes, IL-6 signals *via* the Janus kinase (JAK) pathway to promote C/EBPδ-induced PAI-1 expression (80). In addition, IL-6 signals and activates the IL-6R/signal transducer and activator of transcription 3 (STAT3) pathway (54), which can indirectly upregulate PAI-1 *via* miR-34a (81). TNF-α can also upregulate PAI-1 (82). However, it is less commonly documented in the literature, and the mechanism remains unknown (**Table 5**).

**Table 5:** IL-6 and TNF-α promote the expression of PAI-1.

Promote expression	Cell/host	Model	Possible mechanism	Reference
TNF-α upregulates PAI-1	Clinic patients	Atherosclerosis	TNF-α inhibition with infliximab decreases PAI-1 Ag level	(82)
IL-6 upregulates PAI-1	Clinic patients/HUVECs	Patients diagnosed with CRS from sepsis	Tocilizumab treatment decreased the PAI-1 levels and alleviated critical illness in severe COVID-19 patients	(18, 42)
	Human hepatoma/primary mouse hepatocytes	–	IL-6 induces PAI-1 expression through JAK signaling pathways converging on C/EBPδ	(80)
	Human colorectal cancer/breast cancer/prostate cancer	Rag1^−/−^ PAI1^−/−^/Rag1^−/−^PAI-1 mice (54)	IL-6 activates the IL-6/STAT3 pathway and, through miR-34a, upregulates PAI-1	(54, 81)

**Figure 2:**
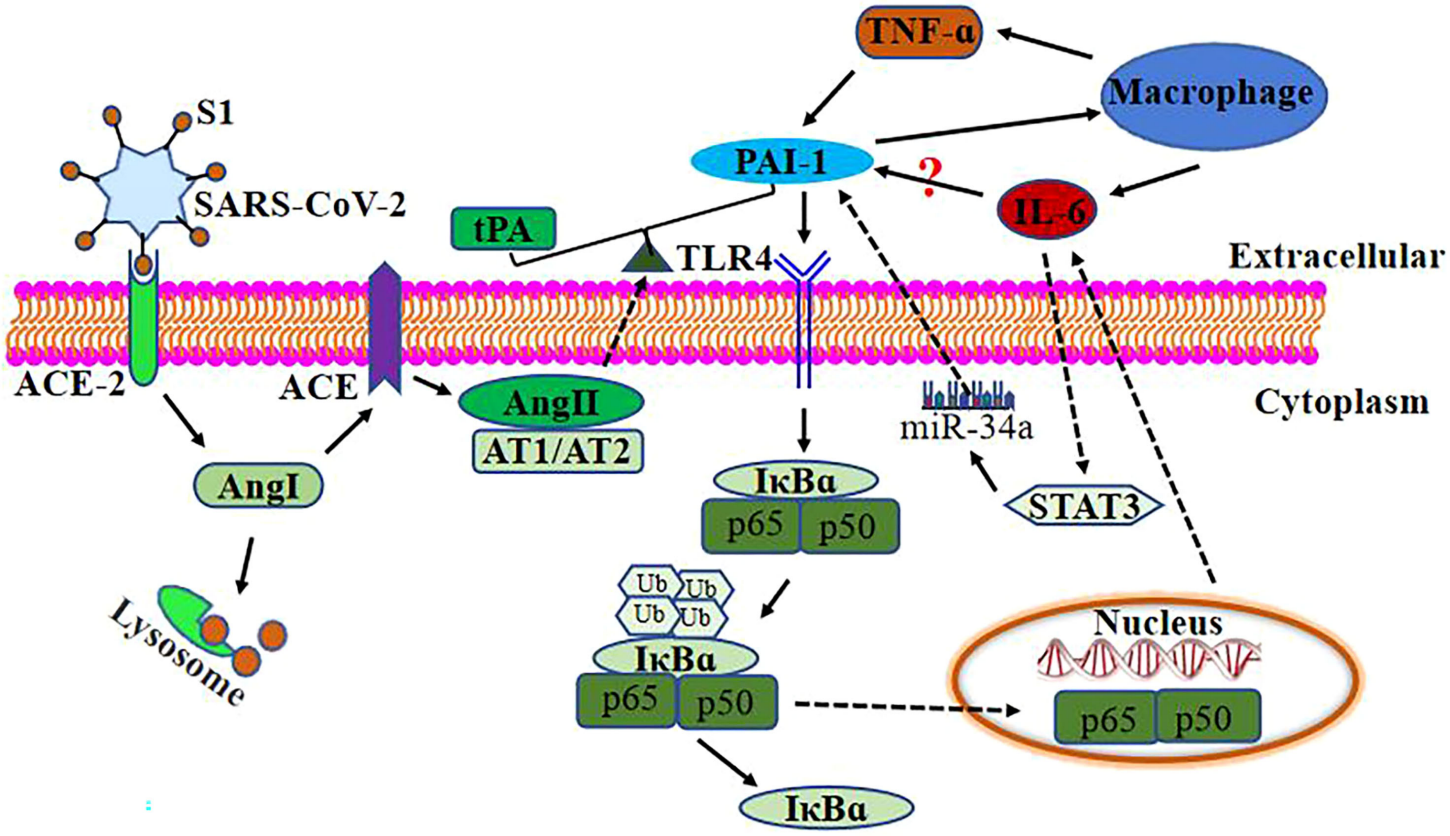
Relationship between PAI-1 and IL-6 after SARS-Co-2 infection. SARS-CoV-2 binds to ACE-2 on the target cell surface, resulting in the loss of ACE-2. ACE-2 is a negative regulator that works by activating tPA. ACE-2 deficiency loses the effective ACE-2/angiotensin (1–7)/Mas receptor axis and increases the level of Ang1. ACE converts Ang I to Ang II and decreases tPA activity, causing endothelial cells and smooth muscle cells to synthesize and release PAI-1. Ang II binds to AT1/AT2 to break the balance of PAI-1/tPA to its prethrombotic state. Elevated levels of PAI-1 in severely and critically ill COVID-19 patients may upregulate IL-6 expression through TLR4/NF-κB pathway and activate macrophages to upregulate IL-6 and TNF-α expression. At the same time, TNF-α can also upregulate PAI-1 expression. IL-6 upregulates the expression of PAI-1 *via* STAT3/miR-29a.

According to the preceding discussion, elevated and persistent IL-6, TNF-α, and PAI-1 levels in severe COVID-19 patients potentially generate a vicious cycle of inflammatory response and thrombosis (**Figure 2**).

## Clinical Significance

The probable inflammatory response and thrombus interaction mechanisms are first described in critically ill COVID-19 patients. TCZ is a recombinant human-resistant human IL-6R IgG1 monoclonal antibody (83). The use of TCZ in critically ill COVID-19 patients can decrease PAI-1 levels and improve the condition of severe COVID-19 patients (42). TCZ is authorized for the treatment of rheumatoid arthritis (84) and systemic juvenile idiopathic arthritis (85) because it selectively binds soluble and membrane-bound IL-6 receptors and inhibits IL-6-mediated classic cis and trans-signaling (86). IL-6 levels in severe COVID-19 patients are significantly higher than in other patients, prompting several researchers to recommend TCZ to inhibit IL-6 signaling in patients with severe COVID-19 to improve patient symptoms (35, 87). According to reports, TCZ can be used as an alternative therapy for COVID-19 patients who are at risk of cytokine storms (88). It is advised that in critically ill patients with elevated IL-6 levels, a repeated dose of TCZ will be necessary to reduce IL-6 levels significantly (88). However, TCZ is ineffective for patients with moderate COVID-19 (89) but can improve clinical symptoms in severely and critically ill COVID-19 patients (90). Breathing and bilateral diffuse turbidity disappear by intravenous TCZ in severe COVID-19 patients with pneumonia and acute respiratory distress syndrome (ARDS) (91). Unfortunately, thrombosis in severe COVID-19 patients was not mentioned. PAI-1 inhibition can improve the level of IL-6 and the damage to ECs. Treatment with TM5614 (PAI-1 inhibitor) eliminates the elevated circulating levels of PAI-1 and thrombin in plasma produced by particulate matter (PM) 2.5 (92). Meanwhile, TM5614 significantly reduces the elevated level of IL-6 (92). Bortezomib, a proteasomal degradation inhibitor, enhances KLF2, decreases PAI-1 expression, and reduces EC damage in HPMECs stimulated with rSARS-CoV-2-S1 glycoprotein (58). PAI-1 may have a role in prothrombotic events and inflammation in COVID-19 patients. This asserts the vicious cycle of PAI-1 and IL-6 in COVID-19.

## Conclusion and Future Perspective

**Figure 3:**
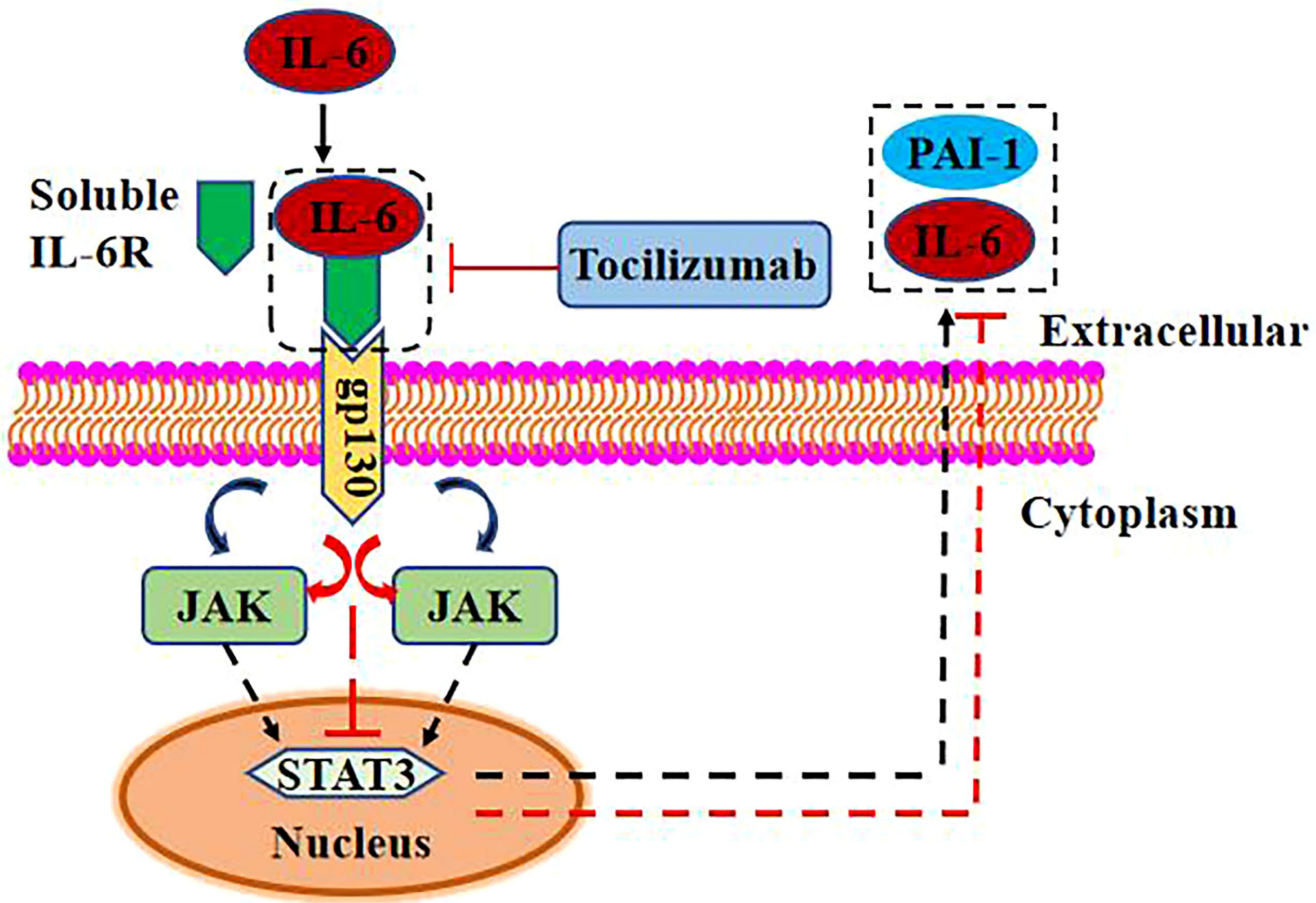
IL-6 promotes PAI-1 expression *via* trans signaling. High concentration of IL-6 combined with soluble IL-6R can activate the JAK/STAT3 signal pathway through gp130 and upregulate the expression of PAI-1 and IL-6. TCZ can reduce the expression of PAI-1 and IL-6 by inhibiting the binding of IL-6 and soluble IL-6R.

In this review, we briefly discussed the possible link between elevated IL-6 levels and thrombosis in COVID-19 patients. From non-viral contexts, the link between PAI-1 and IL-6 forms an inflammatory–thrombus circuit (42). PAI-1 and IL-6 were not shown to be strongly connected in COVID-19 case reports, although autopsy demonstrated substantial damage to ECs (93). In COVID-19 patients, inflammation and thrombosis are two of the most significant deleterious responses (94, 95). The development of blood clots in the heart can be explained by the distribution of ECs in the heart and by the above process (96). In critically ill COVID-19 patients, EC dysfunction increases PAI-1 expression (17) and promotes macrophage recruitment and activation (54). This raises the amount of IL-6 and TNF-α in the blood, increasing the odds of a “cytokine storm” (28). TCZ can decrease IL-6 signal transduction *via* IL-6R and soluble IL-6R. TNF-α, on the other hand, stimulates endothelial PAI-1 production and activates macrophages, exposing ECs to prominent levels of IL-6 and TNF-α and causing sustained tissue and organ damage. In thrombosis, therapeutic use of thrombolytic treatment merely lowers fibrin production. Inability to directly suppress PAI-1 expression and break the vicious cycle between PAI-1 and IL-6 results in serum PAI-1 and IL-6 buildup, facilitating tissue damage and thrombosis development. IL-6 trans-signaling has been shown to increase PAI-1 expression. When IL-6 is coupled with soluble IL-6R and gp130, it activates the downstream JAK/STAT signal pathway and promotes the expression of IL-6 and PAI-1 (54, 97, 98) (**Figure 3**). STAT3-dependent transcription inhibition significantly reduces VEGF-induced vascular permeability in zebrafish, mouse, and human endothelial cells (99). Increased endothelial cell permeability can aggravate pulmonary edema and dyspnea in COVID-19 patients (100). Although the connection between PAI-1 and IL-6 has not yet been shown, the possibility of a malignant interaction between PAI-1 and IL-6 in critically ill COVID-19 patients should not be overlooked. PAI-1 and IL-6 may produce a vicious cycle in which their expression is mutually induced, but the mechanism involved remains unclear. Thrombosis and inflammatory responses in patients with severe COVID-19 are discussed from a new perspective, which provides innovative ideas for future studies.

## Author Contributions

All authors have read and approved the manuscript. FG, YL (11th author), and LY supervised and edited the final manuscript with comments from co-authors. PH, QZ, YL (3rd author), and PO conceptualized and wrote the initial draft, which was further reviewed and edited by FT, YW, XL, JL, and QW for intellectual content. All authors provided crucial revisions in subsequent drafts.

## Funding

This work was supported by the Tianjin Municipal Education Commission Scientific Research Project (Natural Science, Grant No. 2019ZD11 to LY), Science and Technology Program of Tianjin (21ZYJDJC00070), the National Key Research and Development Program of China (2019YFC1708803), and Innovation Team and Talents Cultivation Program of National Administration of Traditional Chinese Medicine (ZYYCXTD-C-202203).

## Conflict of Interest

The authors declare that the research was conducted in the absence of any commercial or financial relationships that could be construed as a potential conflict of interest.

## Publisher’s Note

All claims expressed in this article are solely those of the authors and do not necessarily represent those of their affiliated organizations, or those of the publisher, the editors and the reviewers. Any product that may be evaluated in this article, or claim that may be made by its manufacturer, is not guaranteed or endorsed by the publisher.

## Abbreviations

ACE, Angiotensin-converting enzyme; COVID-19, Coronavirus disease 2019; ECs, Endothelial cells; EGFR, Epidermal growth factor receptor; HFD, High-fat diet; HPMECs, Human pulmonary microvascular endothelial cells; ICU, Intensive care unit; IL, Interleukin; IL-6R, Interleukin-6 receptor; JAK, Janus kinase; LPS, Lipopolysaccharide; MD2, Myeloid differentiation protein 2; NF-kB, Nuclear factor of kappa B; PAI-1, Plasminogen activator inhibitor 1; STAT3, Signal transducer and activator of transcription 3; TCZ, Tocilizumab; TLR, Toll-like receptors; TNF, Tumor necrosis factor; tPA, Tissue plasminogen activator; uPA, Urokinase-type plasminogen activator.
